# Species delimitation of the *Hyphydrus ovatus* complex in western Palaearctic with an update of species distributions (Coleoptera, Dytiscidae)

**DOI:** 10.3897/zookeys.678.12886

**Published:** 2017-06-06

**Authors:** Johannes Bergsten, Elisabeth Weingartner, Jiří Hájek

**Affiliations:** 1 Department of Zoology, Swedish Museum of Natural History, Box 50007, SE-104 05 Stockholm, Sweden; 2 Department of Biology Education, Stockholm University, SE-106 91 Stockholm, Sweden; 3 Department of Entomology, National Museum, Cirkusová 1740, CZ-193 00 Praha 9 – Horní Počernice, Czech Republic

**Keywords:** Dytiscidae, *Hyphydrus*, new records, Palaearctic region, Slovakia, Turkey, Ukraine, GMYC, species delimitation, reciprocal monophyly

## Abstract

The species status of *Hyphydrus anatolicus* Guignot, 1957 and *H. sanctus* Sharp, 1882, previously often confused with the widespread *H. ovatus* (Linnaeus, 1760), are tested with molecular and morphological characters. Cytochrome c oxidase subunit 1 (CO1) was sequenced for 32 specimens of all three species. Gene-trees were inferred with parsimony, time-free bayesian and strict clock bayesian analyses. The GMYC model was used to estimate species limits. All three species were reciprocally monophyletic with CO1 and highly supported. The GMYC species delimitation analysis unequivocally delimited the three species with no other than the three species solution included in the confidence interval. A likelihood ratio test rejected the one-species null model. Important morphological characters distinguishing the species are provided and illustrated. New distributional data are given for the following species: *Hyphydrus anatolicus* from Slovakia and Ukraine, and *H. aubei* Ganglbauer, 1891, and *H. sanctus* from Turkey.

## Introduction

### History of classification

The genus *Hyphydrus* Illiger, 1802 represents a well-defined group of medium sized, globular shaped Dytiscidae. Altogether 139 species occur in all regions of the Old World, with most species distributed in tropical Africa (Miller and Bergsten 2016; Nilsson and Hájek 2017a). A taxonomic revision of the genus was published by Biström (1982).

Only three *Hyphydrus* species occur in Europe (cf. Nilsson and Hájek 2017b). While the Mediterranean *H. aubei* Ganglbauer, 1891 can be easily identified based on black markings on ferrugineous dorsal surface, the uniformly dark-ferrugineously coloured *H. anatolicus* Guignot, 1957 is very similar to the widespread western Palaearctic *H. ovatus* (Linnaeus, 1760) and it was not recognised until 1957. *Hyphydrus anatolicus* was described originally from Angora [= Ankara], Turkey (Guignot 1957). Subsequently Sanfilippo (1963) described the same species under the name *H. carrarai* Sanfilippo, 1963 from Italy. The synonymy of both species was established by Pederzani (1976). The species was later included in the revision of Biström (1982), who synonymized *H. anatolicus* with the older name *H. sanctus* Sharp, 1882, known previously only from the Levant region. Biström (1982) also argued that *H. sanctus* and *H. ovatus* should possibly be regarded as subspecies, but that more work was needed. Although Wewalka (1984) described the differences between *H. anatolicus* and *H. sanctus*, and a habitus photo of *H. anatolicus* was published by Hájek (2009), both mentioned species remain enigmatic, predominantly because of their similarity with *H. ovatus*, and because their distribution is not satisfactorily known.

## Molecular data from museum specimens

With the advance of DNA Barcoding, extraction and amplification techniques have moved forwards in two directions. First towards high-throughput low-cost facilities racing from specimens to barcodes (Ivanova et al. 2006) and boosted by next-generation sequencing techniques (Shokralla et al. 2014). Second towards non-destructively generating DNA sequence data from older museum material with degenerated DNA (Gilbert et al. 2007). The latter will get ever more important as local and global extinction of species due to human activities means that getting fresh material of many species will be impossible or increasingly difficult. Therefore the only resort is to old, often dry-pinned or dry-mounted museum material, with the DNA degraded to various degrees. Little is known about exactly how fast DNA degrades under various conditions (but see Allentoft et al. 2012), but any probability model will have longer half-time the shorter the fragment. Thus, aiming for shorter amplicon size has been the preferred method, not least seen in the field of ancient DNA (Thomsen et al. 2009).

In this study, one of the three focal species is very rarely collected hence we attempt to amplify a >800bp segment of cytochrome c oxidase subunit 1 (CO1), from 19–25 years old dry-mounted specimens. We do this by using additives to standard DNA extraction lysis solutions and designing a number of internal primers to amplify the target segment in six short but overlapping fragments. Extractions are done on whole body but completely non-destructive, an important requirement for invaluable museum specimens.

We also use the general mixed Yule coalescence model (Pons et al. 2006) and a likelihood ratio test to explicitly test whether the *H. ovatus*-complex is better seen as one species (null hypothesis) or several species (alternative hypothesis) in a statistical likelihood framework. The GMYC model was developed as a tool for exploring and delimiting poorly known faunas based on DNA sequences. However here we use it in the context of testing questioned taxa of unsettled taxonomic status in an integrated toolbox where both DNA sequence data, speciation/coalescence models and morphological data bear evidence on the hypothesis.

To clarify the status and distribution of *Hyphydrus anatolicus* and *H. sanctus*, we provide a basal differential diagnosis of both species and related *H. ovatus*. We confirm the specific status of all taxa with molecular analysis. In addition, we review published records and add new faunistic data for *H. anatolicus* and *H. sanctus*, as well as the first record of *H. aubei* from Turkey.

## Material and methods

*Hyphydrus ovatus* was sampled throughout Europe. We acquired fresh material of *H. anatolicus* from Russia and dry-mounted specimens from Turkey, Greece and Slovakia. *Hyphydrus sanctus* was available only as dry-mounted specimens from Israel and Turkey for molecular analysis; *H. aubei* was used as an outgroup in the parsimony and non-clock analyses. The specimens included in this study are deposited in the following institutional collections; for specimens included in molecular analysis, see Table 1.

**Table 1.** Data on extracted specimens, depository, catalogue number and genbank accession number for the CO1 fragment.

Cat. ID	Species	Country	Region	Place	Date	Lat / Lon	Collector	Depository	CO1bp	Acc. No.
704819	*Hyphydrus ovatus*	UK:Scotland	Carrick	Kirkcudbrightshire, Syllodioch	25:VI:2005	55.215N, -4.499W	G.N. Foster	NHM:BMNH	825	FN998871
721926	*Hyphydrus aubei*	Spain	Tarragona	riu Algars, Horta de San Joan	26:V:2005	40.991N, 0.277E	I. Ribera	NHM:BMNH	825	FN998872
722129	*Hyphydrus ovatus*	UK:England	Norfolk	East Harling Common	10:VII:2005	52.451N, 0.942E	G. Nobes	NHM:BMNH	825	FN998873
722301	*Hyphydrus ovatus*	UK:Scotland	Carrick	Boreland of Girthon, Kirkcudbrightshire	02:VII:2005	55.215N, -4.499W	G.N. Foster	NHM:BMNH	788	FN998874
722447	*Hyphydrus ovatus*	UK:England	Norfolk	Thompson Common	03:VII:2005	52.532N, 0.855E	G. Nobes	NHM:BMNH	689	FN998875
724810	*Hyphydrus ovatus*	UK:England	Norfolk	Thompson Common	25:IX:2005	52.529N, 0.854E	G. Nobes	NHM:BMNH	731	FN998876
749803	*Hyphydrus ovatus*	Sweden	Ångermanland	Torrböle	12:VI:2005	63.716N, 19.558E	AN. Nilsson	NHM:BMNH	751	FN998877
729468	*Hyphydrus ovatus*	Sweden	Öland	Borgholm, Langlöt	20:VI:2005	56.747N, 16.685E	J. Geijer	NHM:BMNH	702	FN998878
729591	*Hyphydrus ovatus*	Sweden	Öland	Borgholm, Högsrum	19:VII:2005	56.795N, 16.598E	J. Geijer	NHM:BMNH	825	FN998879
729653	*Hyphydrus ovatus*	Latvia	Riga district	Gaujus National Park, Sigulda, Maza velnala	10:VI:2005	57.152N, 24.865E	L. Hendrich	NHM:BMNH	825	FN998880
729731	*Hyphydrus ovatus*	Latvia	Cesis district	Gaujas National Park, Klamani village	11:VI:2005	57.3N, 25.25E	L. Hendrich	NHM:BMNH	825	FN998881
743243	*Hyphydrus ovatus*	Germany	Bavaria	Eitting, Eittinger Moos	19:VI:2005	48.3N, 11.933E	M. Balke	NHM:BMNH	825	FN998882
743298	*Hyphydrus ovatus*	Germany	Brandenburg	1.5 km N Fresdorf	18:X:2005	52.267N, 13.083E	L. Hendrich	NHM:BMNH	750	FN998883
743957	*Hyphydrus ovatus*	Germany	Bavaria	Murnauer Moos, Rollisch See	06:IX:2005	47.683N, 11.2E	M. Balke	NHM:BMNH	825	FN998884
743962	*Hyphydrus ovatus*	Sweden	Öland	Borgolm, Vanserum	16:VIII:2005	56.691N, 16.641E	J. Geijer	NHM:BMNH	825	FN998885
743968	*Hyphydrus ovatus*	Sweden	Öland	Borgolm, Runsten	07:VII:2005	56.716N, 16.633E	J. Geijer	NHM:BMNH	825	FN998886
743973	*Hyphydrus ovatus*	Sweden	Öland	Mörbylånga, Algustrum	27:VIII:2005	56.687N, 16.598E		J. Geijer	NHM:BMNH	825	FN998887
800099	*Hyphydrus ovatus*	Russia	Volgograd Obl	between Lisov & Polodin	29:IV:2002	48.617N, 43.169E	J. Bergsten	NRM:NHRS	825	FN998888
800100	*Hyphydrus anatolicus*	Russia	Volgograd Obl	between Lisov & Polodin	29:IV:2002	48.617N, 43.169	J. Bergsten	NRM:NHRS	825	FN998889
800104	*Hyphydrus anatolicus*	Russia	Volgograd Obl	between Lisov & Polodin	30:IV:2002	48.617N, 43.169E	J. Bergsten	NRM:NHRS	825	FN998890
800105	*Hyphydrus anatolicus*	Russia	Volgograd Obl	Artyedinsko Donskie Peski	03:V:2002	49.686N, 43.333E	J. Bergsten	NRM:NHRS	825	FN998891
800108	*Hyphydrus ovatus*	Russia	Volgograd Obl	Krasnoslobodsk N.P.	4-5:V:2002	48.7N, 44.6E	J. Bergsten	NRM:NHRS	825	FN998892
800109	*Hyphydrus anatolicus*	Russia	Volgograd Obl	Kretskiy	05:V:2002	48.608N, 44.706E	J. Bergsten	NRM:NHRS	825	FN998893
800115	*Hyphydrus ovatus*	Russia	Volgograd Obl	Baybaev, river Don	8-11:V:2002	49.175N, 44.007E	J. Bergsten	NRM:NHRS	825	FN998894
824863	*Hyphydrus ovatus*	UK:England	Cornwall	The Lizard Hayle Kimbro pool	01:VII:2005	50.255N, -5.242W	D. Bilton	NHM:BMNH	825	FN998895
JLKB241	*Hyphydrus sanctus*	Israel		Hula reserve	21:III:1985	33.103N, 35.609E	M. Jäch	NMPC:ENT	825	FN998896
JLKB242	*Hyphydrus sanctus*	Israel		Talme Elazar	21:IV:1986	32.445N, 34.978E	M. Jäch	NMPC:ENT	825	FN998897
JLKB243	*Hyphydrus sanctus*	Turkey	Mugla	Köycegiz	27:V:1991	36.973N, 28.686E	M. Jäch	NMPC:ENT	147	FN998898
JLKB244	*Hyphydrus sanctus*	Turkey	Mugla	Köycegiz	27:V:1991	36.973N, 28.686E	Schödl	NMPC:ENT	665	FN998899
JLKB518	*Hyphydrus anatolicus*	Turkey	Mugla	Köycegiz	27:V:1991	36.973N, 28.686E	Schödl	NMPC:ENT	825	JX221701
JLKB519	*Hyphydrus anatolicus*	Slovakia	Slov. Mer.	Tvrdosovce, 1 km N of Tvrdosovce	24:IV:2000	48.147N, 18.065E	T. Kopecky	NMPC:ENT	825	JX221702
JLKB520	*Hyphydrus anatolicus*	Greece	Chalkidiki	Sithonia, 2 km S Kalamitsi	12:VIII:2000	39.97N, 23.988E	J. Hotovy	NMPC:ENT	825	JX221703

**BMNH** Natural History Museum [former British Museum (Natural History)], London, Great Britain (Christine Taylor);

**HFCB** Hans Fery collection, Berlin, Germany (property of NHMW);

**NHMW** Naturhistorisches Museum, Wien, Austria (Manfred A. Jäch);

**NHRS** Naturhistoriska Riksmuseet (= Swedish Museum of Natural History), Stockholm, Sweden (Johannes Bergsten);

**NMPC** Národní muzeum, Praha, Czech Republic (Jiří Hájek);

**ZMAS** Zoological Institute, Russian Academy of Science, Sankt Petersburg, Russia (Alexander G. Kirejtshuk).

### Molecular analyses

The extraction protocol was different for the fresh alcohol-material of *H. ovatus* and *H. anatolicus* (from Russia) versus the dry-mounted older material of *H. anatolicus* and *H. sanctus*. The former was extracted in 96-well Wizard SV plates following the manufacturers instructions (Promega). The 3’ end of cytochrome c oxidase subunit 1 (CO1) was amplified with the primers PatDyt or RonDyt (Isambert et al. 2011) and Jerry (Simon et al. 1994) using 1ul of DNA, Bioline Taq and the following cycling conditions: 94° for 2min, 35 to 40 cycles of 94° for 30s, 51–53° for 60s and 70° for 90-120s, and a final extension of 70° for 10 min. PCR products were cleaned with a 96-well Millipore multiscreen plate, sequenced in both directions using a Big Dye 2.1 terminator reaction, and analysed on an ABI 3730 automated sequencer. PatDyt and Jerry were used as sequencing primers. The older dry-mounted specimens were extracted using the QIAamp® DNA Micro Kit (QIAGEN®), following the tissue protocol with the addition of 20ul of DTT (Dithiothreitol)(Sigma-Aldrich). PCR was done with a set of 6 newly designed primer pairs (Table 2) amplifying the complete 825bp CO1 segment in shorter overlapping segments between 147 and 228bp long. We used Ready-ToGo™ PCR beads (Amersham Biosciences) together with 1ul of 10uM of each primer, 2ul of DNA and 21ul water in a 25ul reaction. Cycling conditions started with a 5 min denaturation step at 95°C followed by two cycles of 30 s at 95°C, 30 s at 45°C (first, second and fourth fragments) or 50°C (third, fifth and sixth fragments), and 40 s at 72°C, then two cycles of 30 s at 95°C, 30 s at 43°C or 48°C and 40 s at 72°C, and 39 cycles of 40 s at 95°C, 40 s at 41°C or 46°C, 50 s at 72°C, then a final extension step of 8 min at 72°C. PCR reactions were purified with Exonuclease I and FastAP (Fermentas) in the proportion 1:4, and sequenced with a BigDye™ Terminator ver. 1.1 Cycle Sequencing Kit (Applied Biosystems), cleaned with a DyeEx 96 kit (QIAGEN) and run on an ABI Prism 3100 Genetic Analyzer (Applied Biosystems). Sequences are submitted to Genbank under accession codes FN998871-FN998899 and JX221701- JX221703.

**Table 2.** Newly designed primers (apart from Jerry and PatDyt) used to amplify 825bp of CO1 in 6 overlapping fragments from 11-25 years old, dry-pinned, *Hyphydrus* specimens.

Primer	5’ à 3’	Pair	Length
Jerry	CAACATTTATTTTGATTTTTTGG	1	178bp
Hyp178rw	AATATGCTCGAGTATCAAC	1	
Hyp161fw	GTTGTATGAGCTCATCATATA	2	189bp
Hyp349rw	TAGATGAATTTGCAAGGACTAC	2	
Hyp276fw	AGCTACCCTTCACGGATCTC	3	125bp
Hyp400rw	CATAATGAAAGTGAGCCACTAC	3	
Hyp371fw	GTAGTCCTTGCAAATTCATCT	4	228bp
Hyp598rw	CAGGATAGTCTGAGTAACG	4	
Hyp507fw	TTACAGGACTATCATTAAATTCTA	5	147bp
Hyp653rw	CTCCAATAAATGATATAGTAGATC	5	
Hyp616fw	CTCGACGTTATTCAGACTATCC	6	210bp
Patdyt	TCATTGCACTAATCTGCCATATTAG	6	

Sequences were assembled and edited in Sequencher 4.8 (Gene Codes Corporation) and aligned in ClustalX 2.0 (Larkin et al. 2007) with default settings of 15 as gap opening penalty and 6.66 as gap extension penalty. The alignment contained no gaps. Bayesian analysis was done with MrBayes 3.2.1 (Ronquist et al. 2012). We set up a partitioned model based on 3^rd^ resp. 1^st^+2^nd^ codon positions and applied a HKY+G+I model to each partitions, unlinking statefrequencies, t-ratio, shape and proportion of invariable sites. Partitions were allowed separate rates with a variable rate prior. All other prior and proposal settings were left as default. We ran two separate runs each with four chains (one cold and three incrementally heated) 3 million generations sampled every 1000^th^ generation. First 25% was discarded as burn-in. For the first analysis we used a time-free model and rooted the tree with the outgroup *Hyphydrus aubei*. For the second analysis we excluded the outgroup and instead tested the placement of the root with a clockmodel. We used a Bayes Factor test to assess if the data was compatible with a strict molecular clock or if a relaxed clock should be used. A heuristic parsimony analysis was run in Nona (Goloboff 1999) (hold 10000, Mult*100, hold/10, mult*max*) spawned from Winclada (Nixon 1999-2002). The parsimony analysis was followed by optimising the characters on the most parsimonious tree. This was done to show discrete character support for the three species. We performed a species-delimitation analysis using the general mixed yule coalescence model (GMYC) as implemented in R (R Development Core team 2005) with the package Splits (Ezard et al. 2009; Fujisawa and Barraclough 2013). We tested the null-hypothesis that the *Hyphydrus ovatus*-complex is a single species versus the alternative hypothesis that it consists of more than one species with a likelihood ratio test under the GMYC model. The GMYC method optimizes the likelihood of a single threshold across an ultrametric gene-tree. The threshold defines speciation branches towards the root from the threshold and within-species coalescence branches towards the tips from the threshold. The older branches are modelled with a Yule (speciation) model while the younger branches are delimited into n-groups where each group is modelled with a separate coalescent process model. The maximum likelihood solution of the GMYC model (the likelihood is calculated placing the threshold at each node across the tree) is compared against a model treating the entire gene-tree as a single coalescence (i.e. as a single species) in the likelihood ratio test. We used the ultrametric clock-tree generated above as input to the species delimitation test.

### Morphological observations

The specimens were examined using an Olympus SZX12 stereomicroscope. Measurements were taken with an ocular graticule. Habitus photographs were taken using a Canon MP-E 65mm f/2.8 macro lens with 5:1 optical magnification on bellows attached to a Canon EOS 550D camera. Drawings were made based on photographs taken using an Olympus SZX12 microscope equipped with a Canon EOS 1100D digital camera. Images of the same specimen/structure at different focal planes were combined using Helicon Focus 5.1.19 software. To avoid artefacts due to desiccation of poorly sclerotised parts, the genitalia were illustrated mounted in dimethyl hydantoin formaldehyde resin (DMHF) on the same card as the beetle.

## Results

### Molecular analyses

Amplification was highly successful with the short fragment PCRs of old dry-mounted material (Table 3). The full-length 825bp segment was achieved for the two *H. sanctus* specimens from Israel, 665bp for one of the Turkish specimens, and a 147bp segment of the second Turkish specimen, with three ambiguous base calls. The last specimen also gave a 175bp sequence from primer pair 1 (Table 2) that turned out to be contaminated DNA with closest BLAST hit on Genbank being saccharomycete fungi. This is always a risk when extracting DNA from the whole body of a specimen. All three dry-mounted *H. anatolicus* specimens yielded full-length CO1 sequences.

**Table 3.** Details on the older extracted specimens and the associated DNA data.

Species	GUID NMPC:	Country	Specimen state	Age (years)	Bp	Ambiguous base calls
*Hyphydrus anatolicus*	JLKB000000518	Turkey	Dry-mounted	20	825	0
*Hyphydrus anatolicus*	JLKB000000519	Slovakia	Dry-mounted	11	825	0
*Hyphydrus anatolicus*	JLKB000000520	Greece	Dry-mounted	11	825	0
*Hyphydrus sanctus*	JLKB000000244	Turkey	Dry-mounted	20	665	0
*Hyphydrus sanctus*	JLKB000000241	Israel	Dry-mounted	25	825	0
*Hyphydrus sanctus*	JLKB000000242	Israel	Dry-mounted	24	825	0
*Hyphydrus sanctus*	JLKB000000243	Turkey	Dry-mounted	20	147	3

**Figure 1.**
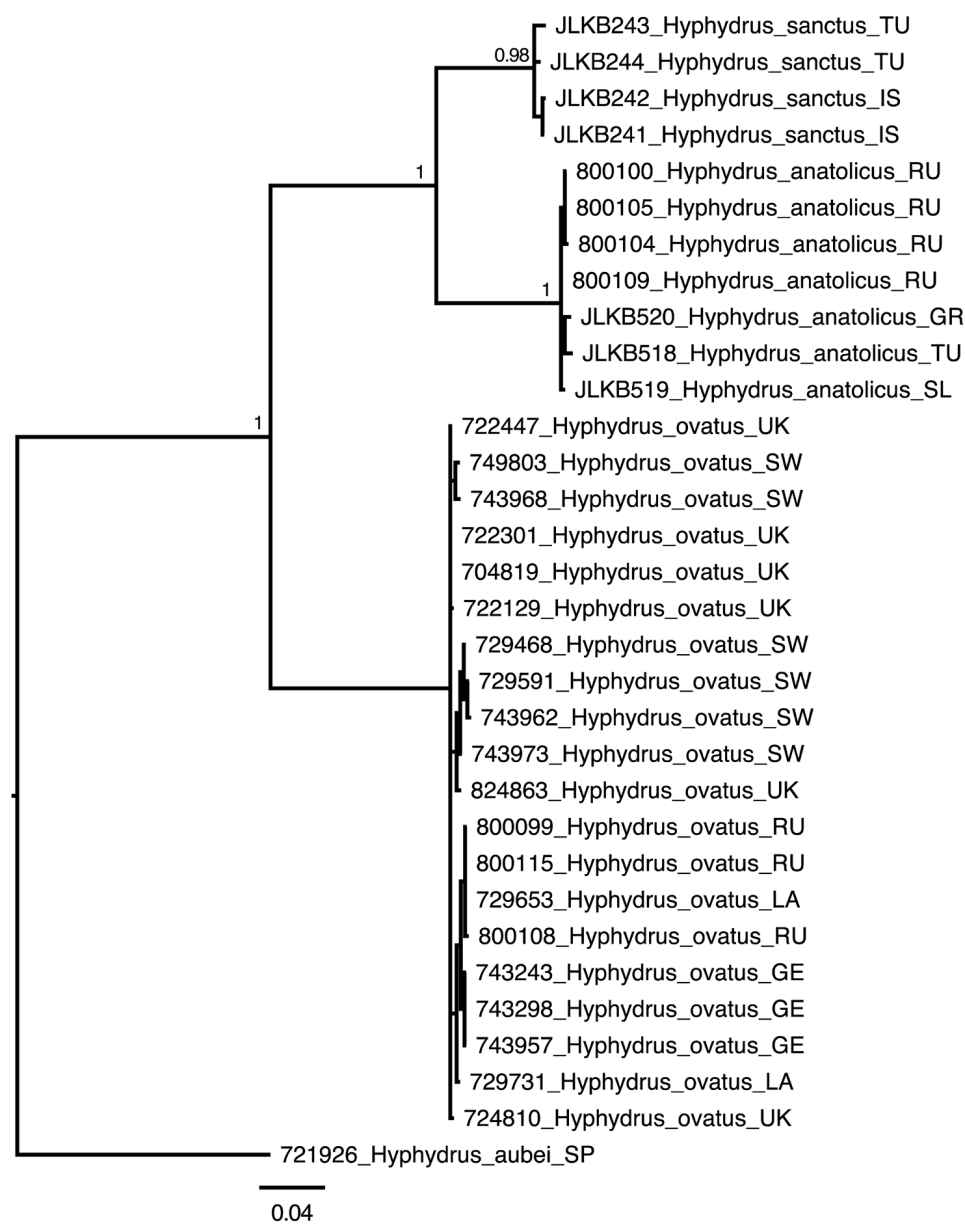
Majority-rule consensus tree from the non-clock Bayesian analysis. Posterior probability clade support values >0.9 shown. Country abbreviations: SW=Sweden, GE=Germany, UK=United Kingdom, La=Latvia, RU=Russia, TU=Turkey, IS=Israel, GR=Greece, SL=Slovakia. Rooted (midpoint) with *Hyphydrus aubei*.

**Figure 2.**
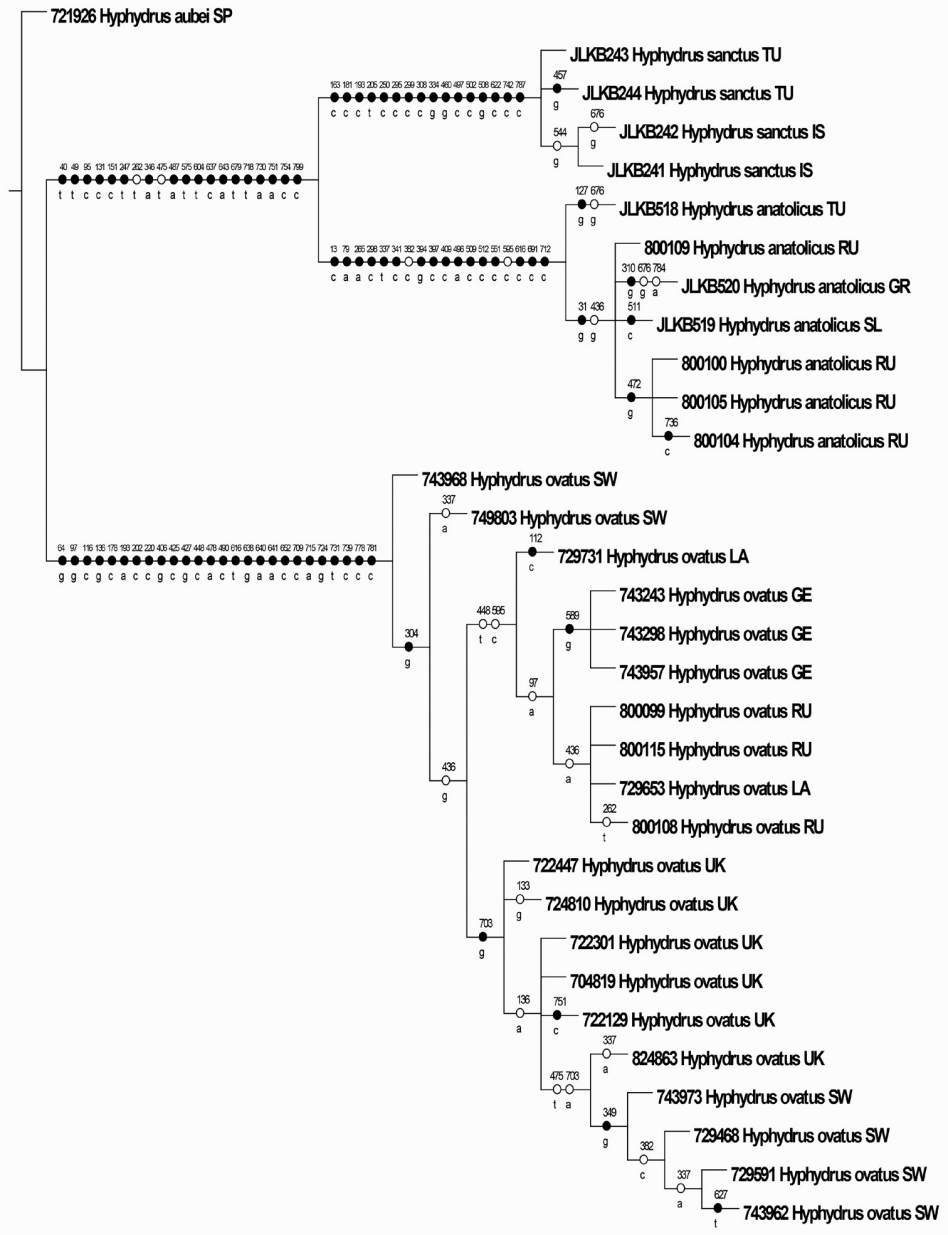
One of 14 most parsimonious trees (L=203, zero-length branches hard-collapsed) with unambiguous characters optimized. Black dots=non-homoplasious characters, white dots=homoplasious characters. Numbers refer to the character’s position in the alignment from 1-825. The other 13 cladograms only differed in within-species internal organizations. Rooted with *Hyphydrus aubei*. Country abbreviations as in Figure 1.

**Figure 3.**
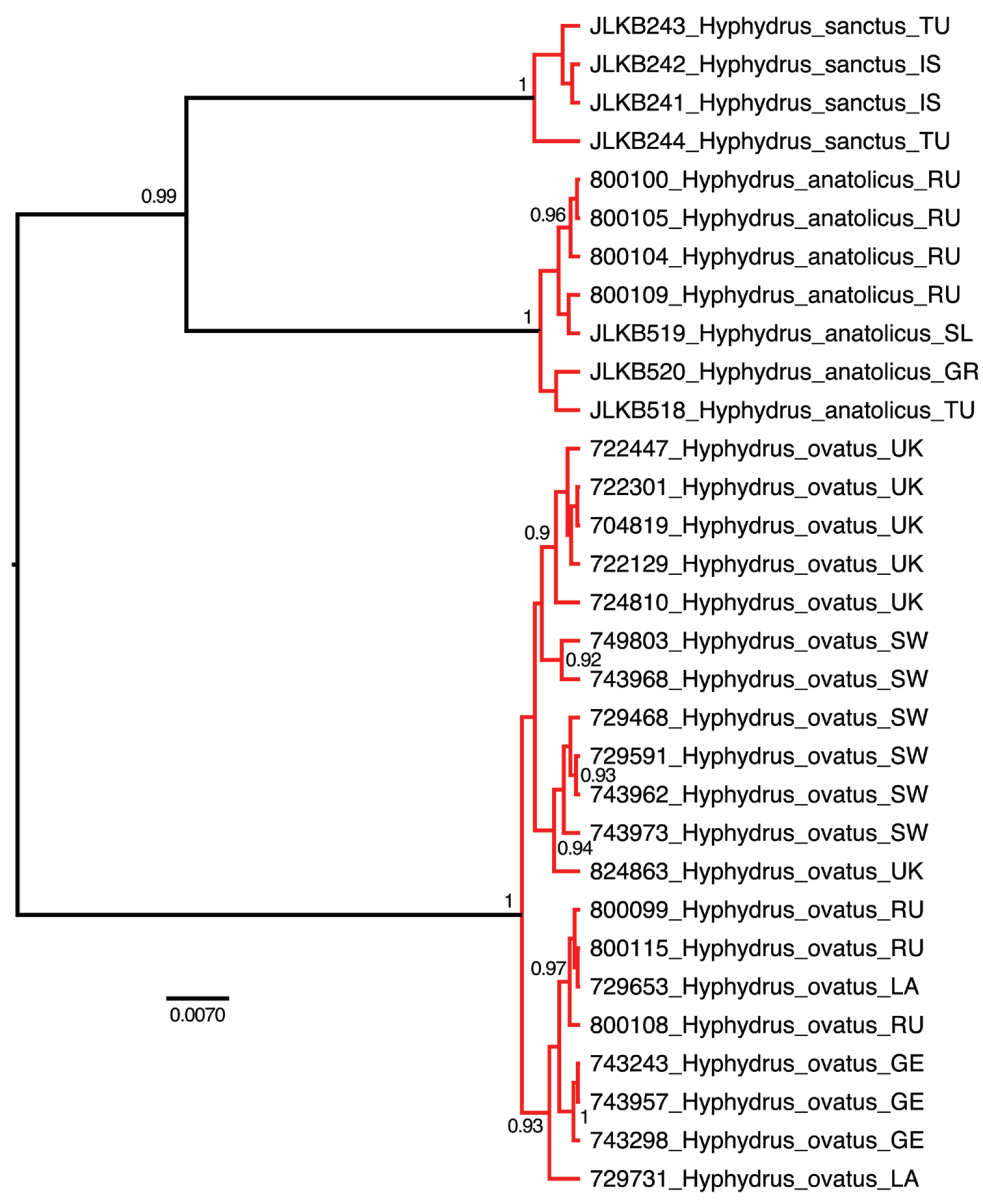
Clock-rooted ultrametric tree from Bayesian analysis with branches coloured according to the GMYC species delimitation analysis. Posterior probability clade support values >0.9 shown. Black branches=speciation events, red braches=within species coalescence events. Country abbreviations as in Figure 1.

Genetic distances between the three presumed species in the *ovatus*-complex turned out to be large (Table 4). The distance between *H. ovatus* and *H. anatolicus* or *H. sanctus* was 9.4–11.4% (K2P-model). The distance between *H. sanctus* and *H. anatolicus* was slightly less, 6.7–7.1%. These genetic distances strongly indicate that we are dealing with three valid and separate species in the *ovatus*-complex. Within-species variation was less than 1.4%. The time-free bayesian analysis as well as the parsimony analysis, both rooted with *H. aubei* as outgroup, confirmed that the three presumed species are reciprocally monophyletic and separated from each other with long branches (Figs 1–2). Posterior probability support values were 1.0–0.98 for all three species. *H. sanctus* and *H. anatolicus* are sister species according to this single-gene phylogeny both in the outgroup-rooted trees (Figs 1–2), and in the clock-rooted tree (Fig. 3). Parsimony analysis and character optimization confirmed the *H. sanctus* + *H. anatolicus* sister group relationship with 17 supporting unambiguous and non-homoplasious substitutions (Fig. 2). Also all three presumed species were supported with between 16 and 24 unambiguous and non-homoplasious substitutions (Fig. 2). The Bayes factor test strongly favoured the strict clock (LnL=-1701) over a time-free model (LnL=-1764) (2*LnBF=125), hence a strict, as oppose to a relaxed, clock model was used to generate an ultrametric tree (Fig. 3). The GMYC model delimited three clusters congruent with the three presumed species as the maximum likelihood solution (Fig. 3). An approximate confidence interval of 2log likelihood units from the maximum likelihood (3 clusters) did not include any other solution. The explicit likelihood ratio test of the null hypothesis of a single coalescing unit (species) was refuted in favour of the alternative hypothesis of three separately evolving and coalescing units (-Log L_one species_= 211.9965, -Log L_three species_= 218.2261, Likelihood ratio=12.4592, p=0.00596).

**Table 4.** Genetic distances between species calculated with Kimura 2-parameter model. Pairwise deletion of missing data was used, and the shortest fragment of *H. sanctus* (147bp) was deleted from comparison.

	*H. ovatus*	*H. anatolicus*	*H. sanctus*	*H. aubei*
*H. ovatus*	0.000–0.014	/	/	/
*H. anatolicus*	0.102–0.114	0.001–0.002	/	/
*H. sanctus*	0.094–0.107	0.067–0.071	0.001–0.008	/
*H. aubei*	0.119–0.126	0.125–0.128	0.132–0.138	-

### Systematics and distribution

All mentioned species belong to the *Hyphydrus ovatus* species group sensu Biström (1982). The group contains nine species occurring exclusively in the Palaearctic region. The members of the group are well characterised with the longer metatibial spur of males serrate (cf. Fig. 7). The four western Palaearctic species share the similar shape of the median lobe of aedeagus which is rather poorly sclerotised, in ventral view nearly parallel-sided with sides straight, very slightly and continually narrowing from base to apex (cf. Fig. 8). Finally, the three species of the *H. ovatus* complex (i.e. *H. anatolicus, H. ovatus* and *H. sanctus*) can be easily recognised by their more or less uniform dark ferrugineous to ferrugineous body colouration, rarely with minor pale markings.

Due to rather weak sclerotisation of external genitalia, the genital characters have only limited use for identification of species in this complex. Therefore, we focused more on habitus, punctuation and structure characters of the species. The most diagnostic character is probably the shape of the longer metatibial spur on males (see Fig. 7). A key to identification of all western Palaearctic species of the *H. ovatus* species group is presented at the end of the taxonomic section.

#### Hyphydrus anatolicus

Taxon classificationAnimaliaColeopteraDytiscidae

Guignot, 1957

Hyphydrus anatolicus Guignot, 1957: 91 (orig. descr.; type locality: “Angora” [Ankara, Turkey]).
Hyphydrus carrarai Sanfilippo, 1963: 77 (orig. descr.; type locality: “Macchia di Migliarino, Torre del Lago (Toscana)” [Italy]); synonymy by Pederzani 1976: 166.
Hyphydrus sanctus : Biström 1982: 39 (partim, misidentification).

##### Published records.

**Bosnia and Hercegovina**: Biström (1982: 39 as *H. sanctus*). **Croatia**: Guéorguiev (1971: 8 as *H. carrarai*); Biström (1982: 39 as *H. sanctus*); Ádám (1992: 194 as *Hyphydrus sanctus*); Temunović et al. (2007: 17); Krčmar (2014: 20). **Greece**: Biström (1982: 39 as *H. sanctus*); Wewalka (1984: 131). **Hungary**: Ádám (1992: 194 as *H. sanctus*); Csabai et al. (1999: 148 as *H. sanctus*); Móra et al. (2004: 153); Csabai and Nosek (2006: 73); Kálmán et al. (2008: 76); Molnár (2008:110); Sóos et al. (2008: 223); Lőkkös (2010:161). **Italy**: Sanfilippo (1963: 77 as *H. carrarai*); Angelini (1972: 182 as *H. carrarai*; 1984: 54); Pederzani (1976: 166); Biström (1982: 39 as *H. sanctus*); Rocchi (1991: 68); Pederzani & Campadelli (1996: 21); Nardi (1997: 132); Bordoni et al. (2006: 87). **Macedonia**: Biström (1982: 39 as *H. sanctus*). **Montenegro**: Scheers (2016: 209). **Russia**: Biström (1982: 39 as *H. sanctus*). **Serbia**: Mesaroš (2015: 50). **Turkey**: Guignot (1957: 91).

##### Material examined.

**Greece**: 2♂♂, Ionian Islands, Kerkyra, Chalikiopoulos [lagoon], 22.iv.1935 (NHMW); 1♂, Eastern Macedonia and Thrace, Évros Distr., plain of Évros river, 26.vii.1988, M. Jäch leg. (NHMW); 1♀, Central Macedonia, Khalkidhiki Distr., Sithonia, 2 km S of Kalamítsion, 12.viii.2000, J. Hotový leg. (NMPC); 5♂♂ 5♀♀, NW Peloponnese, 3 km S Kalogria, 38.1213N, 21.3810E, ca. 3 m, shallow seasonal swamp, 17.v.2010, H. Fery & L. Hendrich leg. (NMPC). **Hungary**: 1♂ 1♀, Hungary 46 36 (BMNH); 1♀, Bács-Kiskun, Kiskunmajsa env., 11.viii.1999, J. Hájek leg. (NMPC). **Montenegro**: 1♂, Vranjina env., Skadarsko jezero, 20.ix.2001, J. Hájek leg. (NMPC). **Russia**: 1♀, Orenburg reg., Totskoye, 1917, Š. Jureček leg. (NMPC); 2♂♂ 2♀♀, Samara, K. Fausta leg. (ZMAS); 1♀, Stavropol reg., Kuma river, 20.iv.1911 (ZMAS); 1♂ 2♀♀, Volgograd reg., 2 km south of Zryanin village, 48°36‘60‘‘N, 43°10‘10‘‘E, small lakes near Liska river, incl silty open bay with grasses, *Alisma* and *Juncus*, 29-30.iv.2002, J. Bergsten & A. Nilsson leg. (NHRS); 1♂ 1♀, Volgograd reg., Archeda-Don rivers alluvial sandy plain, 16 km ESE of Terkin village, 49.6861N, 43.3333E, different lakes, grassy ponds, fens and stream, 2-3.v.2002, J. Bergsten & A. Nilsson leg. (NHRS); 3♂♂ 2♀♀, Volgograd reg., Kretskiy, 48.6083N, 44.7061E, river-arm, newly flooded grassland, 5.v.2002, J. Bergsten & A. Nilsson leg. (NHRS). **Turkey**: 2 spec., Aydin vil. [= province], S of Aydin, ditch, 4.iv.14987, H. Fery leg. (HFCB); 4♂♂ 1♀, Muğla vil. [= province], Köyçeğiz, 27.v.1991, S. Schödl leg. (NHMW, NMPC). **Slovakia**: 1♂, 1 km N of Tvrdošovce, 24.iv.2000, T. Kopecký leg. (NMPC). **Ukraine**: 1♂, Kherson distr., monast. Korsunskij, cursus inf. fl. Dnjepr, 3.vi.1927, S. Medvedev leg. (ex coll. Zakharenko, ZMAS).

##### Diagnosis.

**Figure 4.**
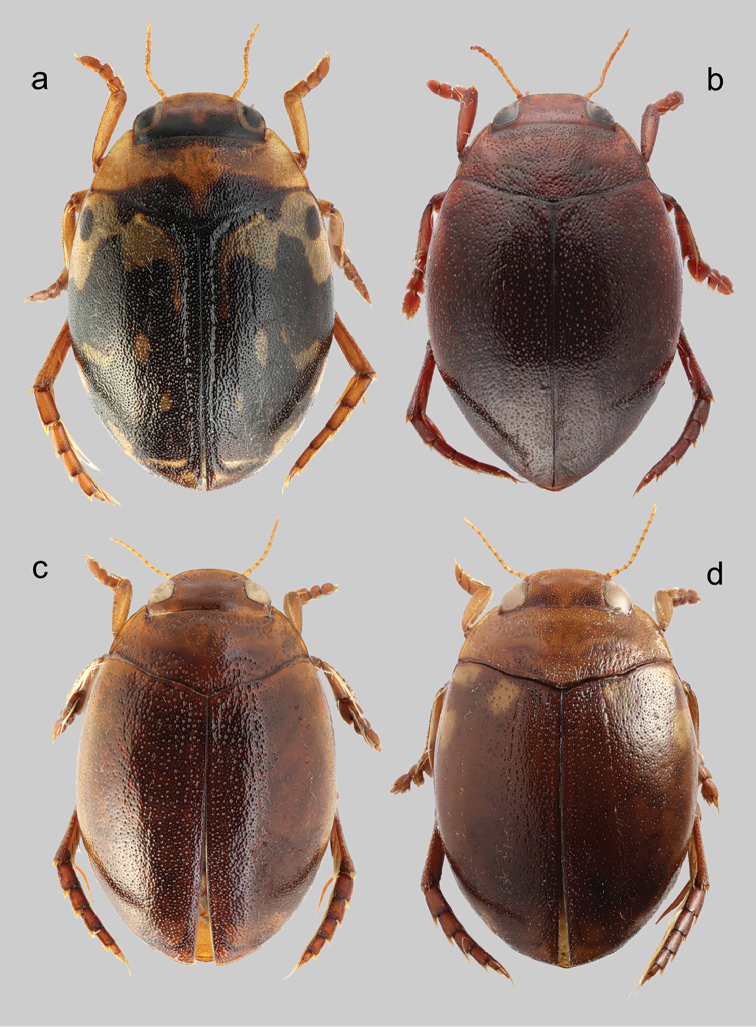
*Hyphydrus* male habitus. **a** *H. aubei* (Corsica; 4.9 mm) **b** *H. ovatus* (Sweden; 5.0 mm) **c** *H. anatolicus* (Slovakia, specimen post-extraction; 5.1 mm) **d** *H. sanctus* (Turkey; 5.2 mm).

**Figure 5.**
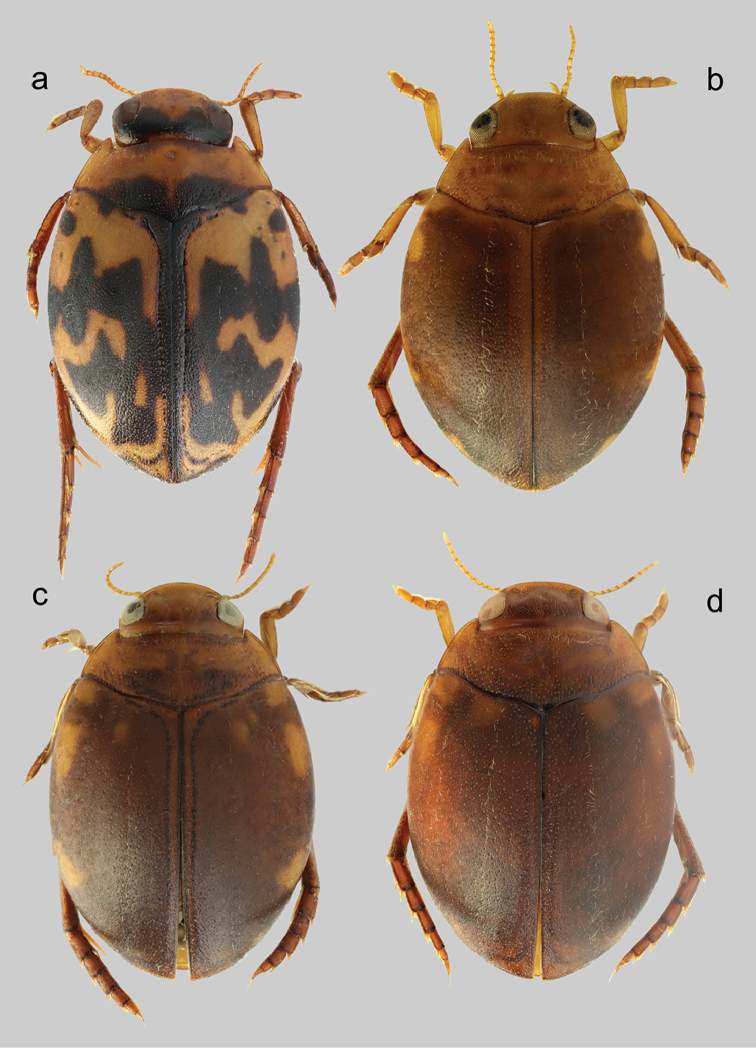
*Hyphydrus* female habitus. **a** *H. aubei* (Croatia; 4.7 mm) **b** *H. ovatus* (Bohemia; 4.6 mm) **c** *H. anatolicus* (Greece; 5.0 mm) **d** *H. sanctus* (Turkey; 4.9 mm).

**Figure 6.**
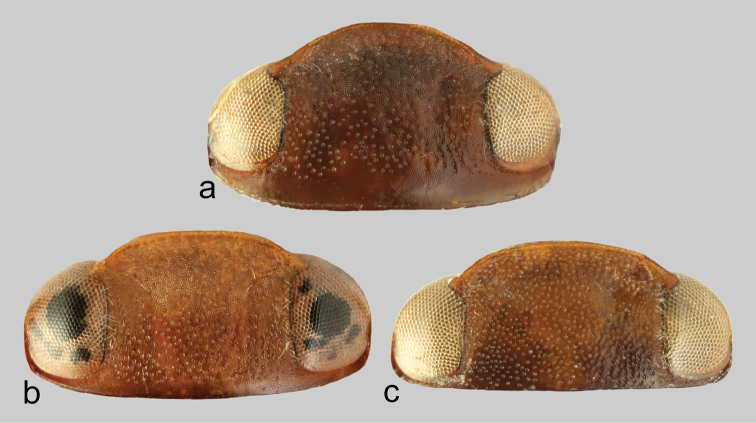
*Hyphydrus* head. **a** *H. anatolicus* **b** *H. ovatus* **c** *H. sanctus*. Not in scale.

Habitus as depicted in Figs 4c, 5c. Clypeus with anterior margin rounded (Fig. 6a). Reticulation of dorsal surface confined to head, more distinct and impressed anteriorly. Punctation of head fine, visible on whole surface; punctures sparse, distance between them usually equal or bigger than their diameter (Fig. 6a). Punctation of pronotum double, fine, distance between larger punctures bigger than their diameter. Punctation of elytra double, diameter of small puncture less than half of diameter of large punctures; distance between large punctures bigger than their diameter. Epipleura smooth with fine punctures. Metatibia with sinuous outer margin.

**Figure 7.**
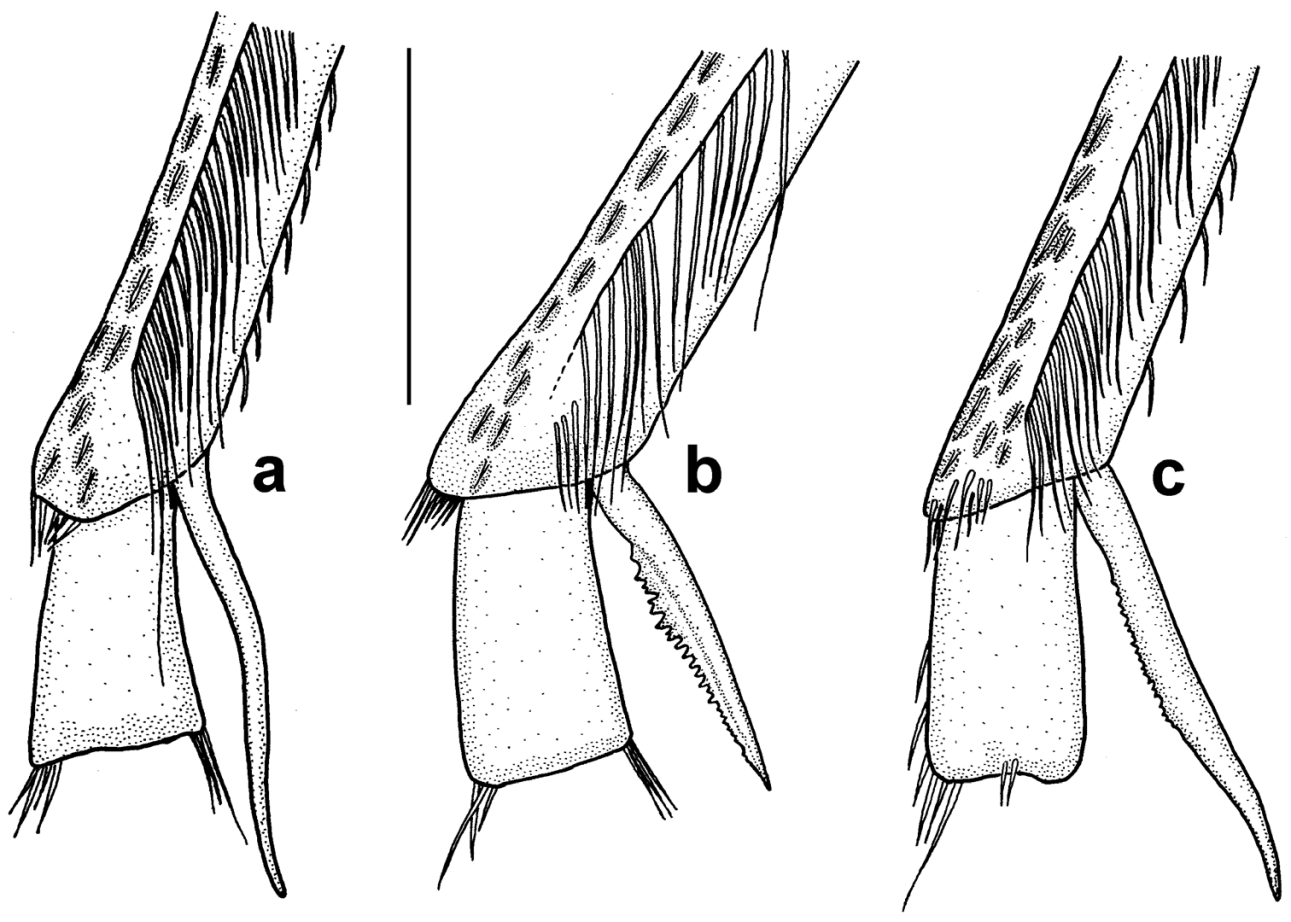
*Hyphydrus* male metatibia, longer metatibial spur and metatarsomere I. **a** *H. anatolicus* **b** *H. ovatus* **c** *H. sanctus*. Scale bar 0.5 mm.

**Figure 8.**
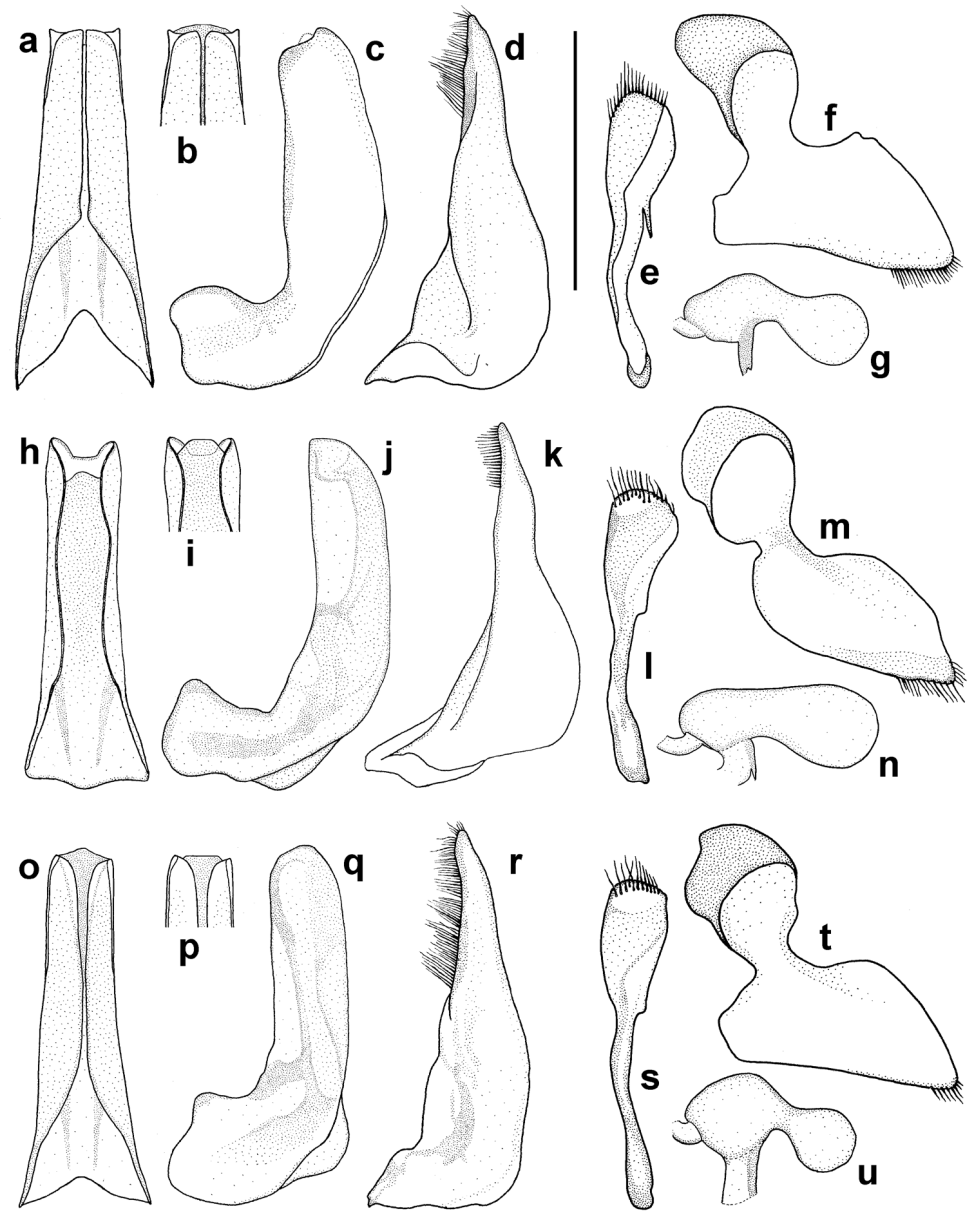
*Hyphydrus* male and female genitalia. **a, h, o** median lobe of aedeagus in ventral view **b, i, p** supplementary drawing of apex of median lobe **c, j, q** median lobe of aedeagus in lateral view **d, k, r** paramere **e, l, s** gonocoxa **f, m, t** gonocoxosternite **g, n, u** spermatheca**. a–g** *H. anatolicus* **h–n** *H. ovatus* **o–u** *H. sanctus*. Scale bar 0.5 mm.

Male. Longer metatibial spur long, nearly as long as metatarsomere I-II combined (Fig. 7a); spur bisinuate with only indistinct serration basally (Fig. 7a). Male genitalia as in Fig. 8a–d, median lobe in ventral view slightly narrowing from base to apex.

Female. Both shiny and matt forms known of females of *H. anatolicus*. Shiny form agreeing well with male; matt form with whole surface densely reticulated, meshes somewhat elongate on elytra. Large punctures well visible, small punctures indistinct among reticulation. Longer tibial spur shorter than in male; broad and with serration in basal two thirds, narrowed, slightly curved and without serration in apical third. Female genitalia as in Fig. 8e–g.

##### Habitat.

The species inhabits various types of standing water, predominantly densely vegetated pools, ditches and small ponds. *H. anatolicus* tolerates also saline habitats.

##### Distribution.

**Figure 9.**
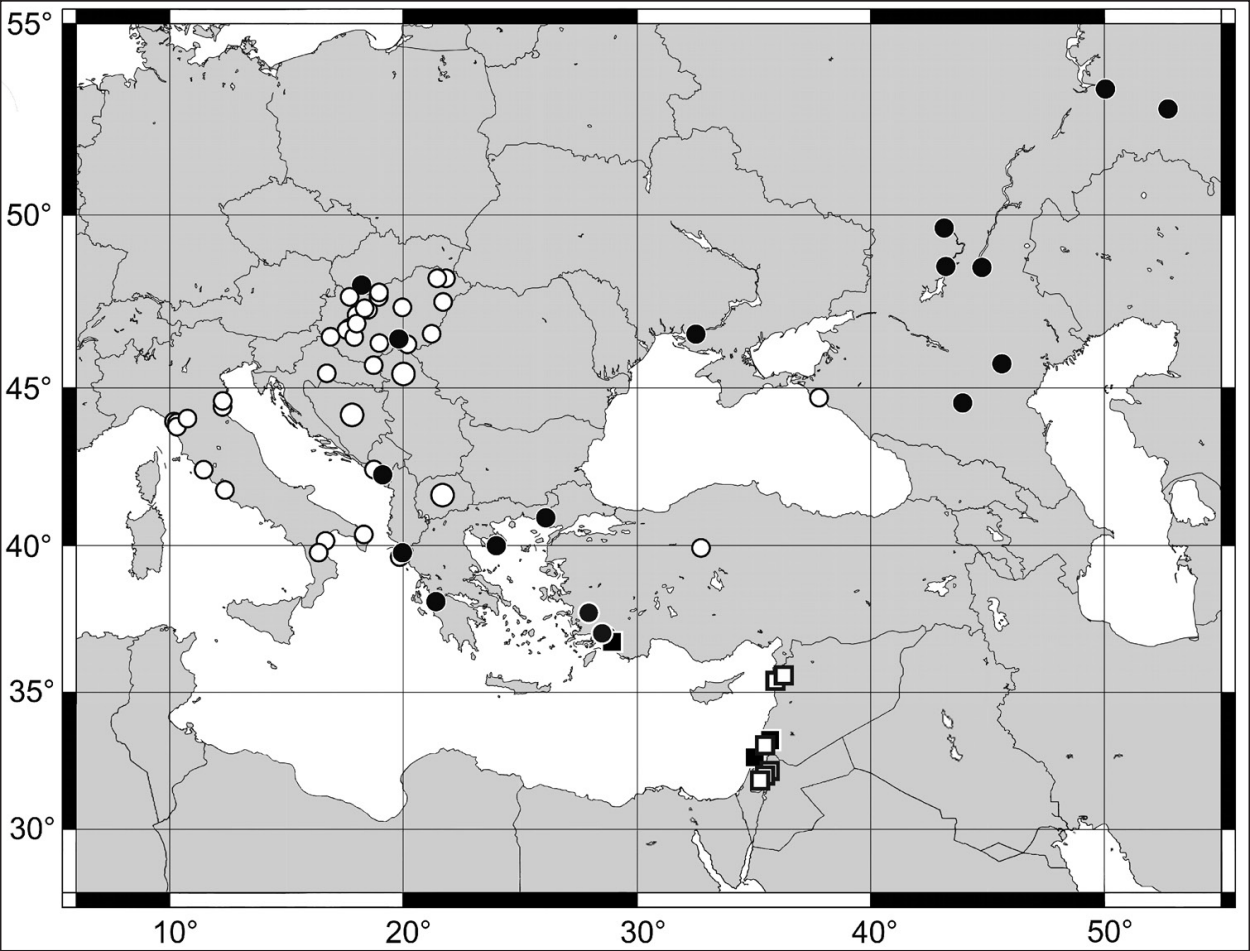
Map of distribution of *H. anatolicus* (circles, dots) and *H. sanctus* (squares). White symbols represent records from the literature, large circles represent imprecise data for a larger region (country); black symbols represent records of specimens examined by us.

The species is distributed in the Eastern Mediterranean and in south-eastern Europe. It occurs in Italy, southernmost Slovakia, Hungary, the Balkan Peninsula, Turkey, southern Ukraine and Russia up to latitude 55° and east to the Ural Mountains (Fig. 9). First record from Slovakia and Ukraine.

#### Hyphydrus ovatus

Taxon classificationAnimaliaColeopteraDytiscidae

(Linnaeus, 1760)

Dytiscus ovatus Linnaeus, 1760: 547 (type locality: Svecia [Sweden]). For full list of synonymy, see Nilsson & Hájek (2017a: 199).

##### Material examined.

We have examined more than 600 specimens from the Czech Republic, Finland, France, Germany, Great Britain, Russia, Slovakia, Sweden, and Ukraine, deposited in NHRS and NMPC.

##### Diagnosis.

Habitus as depicted in Figs 4b, 5b. Clypeus with anterior margin medially nearly straight (Fig. 6b). Reticulation of dorsal surface confined to head, more distinct and impressed anteriorly (Fig. 6b). Punctation of head fine, visible only in posterior half, punctures on clypeus imperceptible due to strong reticulation (Fig. 6b); punctures dense, distance between them smaller than their diameter (Fig. 6b). Punctation of pronotum double, coarse, distance between larger punctures smaller than their diameter. Punctation of elytra double, diameter of small puncture about half of diameter of large punctures; distance between large punctures, at least basally, smaller than their diameter. Epipleura smooth with fine punctures. Metatibia with outer margin nearly straight.

Male. Longer metatibial spur short, only slightly longer than metatarsomere I (Fig. 7b); spur nearly straight, broad with distinct serration (Fig. 7b). Male genitalia as in Fig. 8h–k, median lobe in ventral view parallel-sided in most of its length.

Female. Both shiny and matt forms are known for females of *H. ovatus*. Shiny form agreeing well with male; matt form with whole surface densely reticulated, meshes distinctly elongate on elytra. Large punctures well visible, small punctures indistinct among reticulation. Longer tibial spur similar to that of male. Female genitalia as in Fig. 8l–n.

##### Habitat.

The species inhabits various types of standing and slowly flowing water bodies with at least some vegetation. The typical habitats represent (frequently eutrophic) ponds, densely vegetated pools, ditches, oxbows or open swamps.

##### Distribution.

Widely distributed Palaearctic species. With the exception of the Iberian Peninsula, it occurs in most of territory of Europe and temperate Asia east to the Baikal Lake (east Siberia).

#### Hyphydrus sanctus

Taxon classificationAnimaliaColeopteraDytiscidae

Sharp, 1882

Hyphydrus sanctus Sharp, 1882: 380.

##### Published records.

**Israel**: Sharp (1882: 380); Biström (1982: 39); Wewalka (1984: 131). **Jordan**: Biström (1982: 39); Wewalka (1984: 131). **Syria**: Biström (1982: 39); Wewalka (1984: 131).

##### Material examined.

**Israel**: 2♂♂, 1♀, Hula reserve, 21.iii.1985; 4♂♂, 8♀♀, same locality, but 13.iv.1986; 1♂, 3♀♀, Talme Elazar, 21.iv.1986; 1♂, 6♀♀, Magan Michael, 21.iv.1986, all M. Jäch leg. (NHMW, NMPC). **Turkey**: 3♂♂, 13♀♀, Muğla vil. [=province], Köyçeğiz, 27.v.1991, S. Schödl leg. (NHMW, NMPC); 3♂♂, 2♀♀, same data, but M. Jäch leg. (NHMW, NMPC).

##### Diagnosis.

Habitus as depicted in Figs 4d, 5d. Clypeus with anterior margin medially nearly straight (Fig. 6c). Reticulation of dorsal surface confined to head and more distinct and impressed anteriorly (Fig. 6c), and to sides of pronotum. Punctation of head fine, visible on whole surface (Fig. 6c); punctures dense, distance between them smaller than their diameter (Fig. 6c). Punctation of pronotum double, fine, distance between larger punctures bigger than their diameter. Punctation of elytra double, diameter of small punctures less than half of diameter of large punctures; distance between large punctures bigger than their diameter. Epipleura reticulated with very fine punctures. Metatibia with outer margin nearly straight.

Male. Longer metatibial spur long, nearly as long as metatarsomere I-II combined (Fig. 7c); spur broad and straight in basal two thirds with small but distinct serration, attenuated and curved apically (Fig. 7c). Male genitalia as in Fig. 8o–r, median lobe in ventral view slightly narrowing from base to apex.

Female. Only matt females of *H. sanctus* are known so far. Whole surface densely reticulated, meshes on elytra somewhat elongate. Large punctures well visible, small punctures indistinct among reticulation. Longer tibial spur similar to that of male, but almost straight in apical third. Female genitalia as in Fig. 8s–u.

##### Habitat.

Similarly to the other two species, *H. sanctus* inhabits various types of standing and slowly flowing water bodies with at least some vegetation. Wewalka (1984) reported several specimens from a densely vegetated pool and single occurrences from an artificial pool with clear water, an irrigation ditch and from a stream.

##### Distribution.

A species distributed in the Levant region of the Near East. So far recorded from several localities in Israel, Jordan and Syria (Fig. 9). First record from Turkey.

#### Hyphydrus aubei

Taxon classificationAnimaliaColeopteraDytiscidae

Ganglbauer, 1891

##### Note.

*Hyphydrus aubei* is the fourth European species in the *Hyphydrus ovatus* species group sensu Biström (1982). It does not belong to the *Hyphydrus ovatus* complex as here defined and it is easily separated from the preceding three species based on colouration (Figures 4–5).

##### Material examined.

**Turkey**: 1♂, 2♀♀, Muğla vil. [=province], Köyçeğiz, 27.v.1991, S. Schödl leg. (NHMW, NMPC).

##### Distribution.

Predominantly a Mediterranean species. First record from Turkey.

### Key to species

Key to western Palearctic species of the *Hyphydrus ovatus* species group

**Table:** 

1	Elytra with distinct black maculate colour pattern on elytra; head bicoloured, testaceous anteriorly but distinct black areas posteriorly (Figs 4a, 5a)	***Hyphydrus aubei***
–	Elytra unicoloured, dark ferrugineous to ferrugineous, or with vaguely delimited lighter macula basally and laterally on elytra; head unicoloured, testaceous to dark ferrugineous (Figs 4b–d, 5b–d)	**2**
2	Punctation of pronotum and elytra (males and shiny females) very coarse; distance between larger punctures smaller than their diameter. Longer male metatibial spur only little longer than metatarsomere I; straight and with distinct serration (Fig. 7b)	***Hyphydrus ovatus***
–	Punctation of pronotum and elytra (males and shiny females) finer; distance between larger punctures larger than their diameter. Longer male metatibial spur almost as long as metatarsomeres I-II combined; spur not straight, bisinuate or apically curved; serration of spur small to indistinct (Fig. 7a, c)	**3**
3	Clypeus with anterior margin medially nearly straight; exterior side of metatibia almost straight; longer male metatibial spur straight basally but curved apically and with serration small but visible (Fig. 7c)	***Hyphydrus sanctus***
–	Clypeus with anterior margin rounded; exterior side of metatibia somewhat sinuous; longer male metatibial spur bisinuate and with indistinct serration basally (Fig. 7a)	***Hyphydrus anatolicus***

## Discussion

Our findings from molecular and morphological data unambiguously support the presence of three species of the *Hyphydrus ovatus* complex in the western Palaearctic and the names *H. ovatus*, *H. anatolicus* and *H. sanctus* are the oldest available names for these three species. The additional distributional findings of *H. anatolicus* and *H. sanctus* indicate, that the distribution of the *H. ovatus* complex is more complex in the eastern part of its range than previously thought. A revision of all previous records of *H. ovatus* from the Balkan Peninsula and further east is needed. It is highly probable that many records may refer to the other two species, but whether *H. ovatus* is replaced by, or sympatric with, these remain to be investigated for many areas.

## Supplementary Material

XML Treatment for Hyphydrus anatolicus

XML Treatment for Hyphydrus ovatus

XML Treatment for Hyphydrus sanctus

XML Treatment for Hyphydrus aubei
